# Associations of Physical Activity Level and Variability With 6-Month Weight Change Among 26,935 Users of Connected Devices: Observational Real-Life Study

**DOI:** 10.2196/25385

**Published:** 2021-04-15

**Authors:** Douae El Fatouhi, Lidia Delrieu, Catherine Goetzinger, Laurent Malisoux, Aurélie Affret, David Campo, Guy Fagherazzi

**Affiliations:** 1 Center of Research in Epidemiology and Population Health, UMR 1018 INSERM Institut Gustave Roussy Paris-Sud Paris-Saclay University Villejuif France; 2 Residual Tumor & Response to Treatment Laboratory (RT2Lab), U932 Immunity and Cancer INSERM Institut Curie Paris France; 3 Department of Population Health Luxembourg Institute of Health Strassen Luxembourg; 4 Faculty of Science, Technology and Medicine University of Luxembourg Luxembourg Luxembourg; 5 Withings Issy-les-Moulineaux France

**Keywords:** connected devices, Withings, physical activity, step count, wearable activity trackers, digital health, free-living, weight loss, digital scale, mobile phone

## Abstract

**Background:**

Physical activity (PA) is a modifiable lifestyle factor that can be targeted to increase energy expenditure and promote weight loss. However, the amount of PA required for weight loss remains inconsistent. Wearable activity trackers constitute a valuable opportunity to obtain objective measurements of PA and study large populations in real-life settings.

**Objective:**

We aim to study the associations of initial device-assessed PA characteristics (average step counts and step count variability) and their evolution with 6-month weight change.

**Methods:**

We analyzed data from 26,935 Withings-connected device users (wearable activity trackers and digital scales). To assess the initial PA characteristics and their 6-month changes, we used data recorded during the first and sixth 30-day periods of activity tracker use. For each of these periods, we used the monthly mean of daily step values as a proxy for PA level and derived the monthly coefficient of variation (CV) of daily step values to estimate PA level variability. Associations between initial PA characteristics and 6-month weight change were assessed using multivariable linear regression analyses controlled for age, sex, blood pressure, heart rate, and the predominant season. Restricted cubic spline regression was performed to better characterize the continuous shape of the associations between PA characteristics and weight change. Secondary analyses were performed by analyzing the 6-month evolution of PA characteristics in relation to weight change.

**Results:**

Our results revealed that both a greater PA level and lower PA level variability were associated with weight loss. Compared with individuals who were initially in the sedentary category (<5000 steps/day), individuals who were low active (5000-7499 steps/day), somewhat active (7500-9999 steps/day), and active (≥10,000 steps/day) had a 0.21-kg, a 0.52-kg, and a 1.17-kg greater decrease in weight, respectively (95% CI −0.36 to −0.06, −0.70 to −0.33, and −1.42 to −0.93, respectively). Compared with users whose PA level CV was >63%, users whose PA level CV ranged from 51% to 63%, 40% to 51%, and was ≤40%, had a 0.19-kg, a 0.23-kg, and a 0.33-kg greater decrease in weight, respectively (95% CI −0.38 to −0.01, −0.41 to −0.04, and −0.53 to −0.13, respectively). We also observed that each 1000 steps/day increase in PA level over the 6-month follow-up was associated with a 0.26-kg (95% CI −0.29 to −0.23) decrease in weight. No association was found between the 6-month changes in PA level variability and weight change.

**Conclusions:**

Our results add to the current body of knowledge that health benefits can be observed below the 10,000 steps/day threshold and suggest that not only increased mean PA level but also greater regularity of the PA level may play important roles in short-term weight loss.

## Introduction

### Background

The prevalence of overweight and obesity is continuously increasing; in 2016, 52% of adults worldwide were overweight or obese [1]. Excess body weight, including overweight and obesity, has been associated with an increased risk of several chronic diseases such as cardiovascular diseases, type 2 diabetes, and some types of cancer [2,3] and thus represents a major public health priority. Therefore, weight loss represents a key leverage to prevent or manage numerous obesity-related conditions. Individuals who are overweight and obese are strongly encouraged to attempt at least clinically significant weight loss (≥5% of baseline body weight) [4,5].

Studies have shown that physical activity (PA) has multiple health-related benefits, especially among those who are overweight and obese [6-11]. PA recommendations promote the accumulation of at least 150 minutes per week of moderate-to-vigorous PA for maintaining and improving health. These PA guidelines can also be expressed in terms of steps per day. The most popular translation of existing PA guidelines into steps per day equivalents stated that adults are encouraged to accumulate 10,000 steps per day, 3 to 4 days a week [12,13]. However, the scientific literature on the minimal requirement of PA for weight loss remains to be inconsistent. Although some previous studies suggest that engaging in a PA program following the current public health guidelines may result in no weight loss or modest weight loss in individuals who are overweight and obese [14,15], others have shown that substantial weight loss could still occur if the overall amount of PA far exceeds the minimum recommended level of 150 minutes per week in moderate-to-vigorous PA intensity, particularly by surpassing at least a PA volume of 225 minutes per week [13-16]. In addition, most studies have evaluated the effects of the total amount of PA, but little is known about the best combination of total volume and regularity of PA practice over time.

Monitoring PA over a long period is challenging, especially in large and real-life observational cohort studies. Studies on the associations between PA and weight loss are mostly based on self-reported assessments of both variables. It is well known that self-reported data are prone to social desirability and memory recall bias [17,18]. This often results in overreporting of typical PA habits [19]. Consumer-based wearable activity trackers therefore constitute a valuable opportunity to obtain, over large periods, more objective and precise measurements of PA characteristics. Wearable activity trackers are small, affordable devices that are largely commercially available and allow individuals to objectively and continuously monitor their PA levels by providing real-time feedback [20]. In addition, self-tracking of weight using connected Wi-Fi scales represents a better alternative than self-reported weight values, as it avoids some bias. Data on weight self-monitoring collected from smart scales can also open new research perspectives, including the study of weight loss and weight management among larger populations of real-world users [21,22]. However, these data remain to be underexploited for clinical and epidemiological research purposes.

### Objectives

Therefore, based on a large international population of more than 25,000 users of connected activity trackers and digital scales, we first aim to study the associations of device-assessed PA characteristics (mean PA level and variability in PA level) with weight change over a 6-month period. The secondary aim of this study is to analyze the associations between the evolution of PA characteristics over time and the 6-month weight change.

## Methods

### Study Sample and Available Data

**Figure 1:**
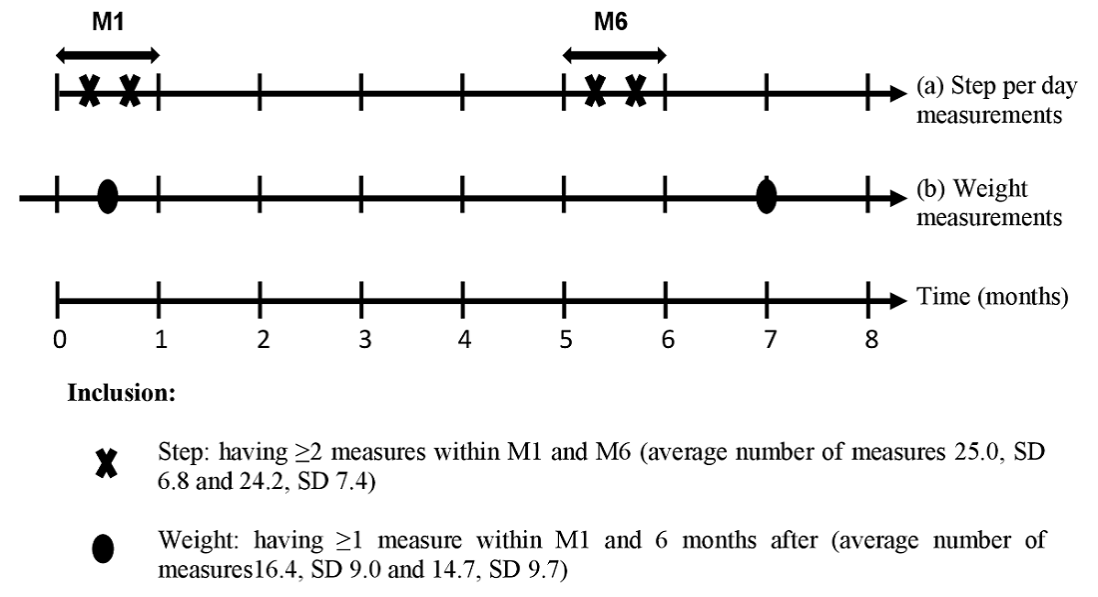
Follow-up and inclusion criteria of the study (N=26,935). (a) Step per day measurements were obtained through the Withings Pulse activity tracker. (b) Weight was assessed by users on their Withings weight scale. M1: first 30-day period of activity tracker use; M6: sixth 30-day period of activity tracker use.

The population was initially composed of a sample of 35,841 highly connected Withings customers, those who had purchased at least three Withings-connected devices: a pulse activity tracker [23,24], a body weighing scale [25], and a BP-800 blood pressure monitor [26] (Withings SA). Customer data, including sex, age, number of steps per day, body weight, heart rate, and blood pressure, were provided by Withings and were collected between October 2009 and April 2016. Data were preprocessed before analysis, as we excluded some outliers from daily data on weight and steps with unlikely values as follows: (1) a lower threshold of 500 steps per day was used to denote valid days with daily step count data [27,28], but the application of this criterion did not result in any deletion of data as the minimum daily step value was 501 steps per day; (2) daily step values exceeding the 99^th^ percentile of the steps per day distribution were removed (99th percentile=22,414 steps/day; 151,937 measurements removed); and (3) weight values lower than 45 kg and above 200 kg were excluded (12,403 measurements removed). In addition, individuals were excluded if they did not (1) have at least two step measurements within the first and sixth 30-day period of activity tracker use (n=4733) and (2) have at least one weight measurement within the first 30-day period of activity tracker use and 6 months after (n=4173). Figure 1 shows the inclusion criteria of this study. The final study sample comprised 26,935 individuals (23,580 males and 3355 females). Consent for participation in this study was obtained electronically when users created their Withings account and accepted the treatment of personal data anonymously by Withings for research purposes.

### PA Assessment

#### Monitoring of PA

The Withings Pulse activity tracker is a commercially available device that can be worn on the wrist or clipped onto a belt or clothing. This activity tracker monitors physical behaviors including the total number of steps taken per day. In addition, it provides the user with real-time feedback on their health metrics through the tracker itself or via an app on the user’s smartphone or tablet with internet access. The wearable device and the app are synchronized, which allows the data recorded by the activity tracker to be instantly transferred from the device to the app via Bluetooth, thus enabling long-term data tracking by users themselves or, for instance, by researchers or health professionals. Wearable activity trackers are considered to be an accurate and objective assessment method that permits PA monitoring [29]. In particular, the Withings Pulse activity tracker has been shown to be reliable and valid for measuring the number of steps taken by healthy subjects under free-living conditions [24,29-31].

#### Assessment of PA Characteristics

To assess the initial PA characteristics and the 6-month change in PA characteristics, we used data recorded during the first and sixth 30-day periods of activity tracker use (named *M1* and *M6* in the following text, respectively). We computed for each of these periods: (1) the mean daily steps and (2) the coefficient of variation (CV) of daily steps. The information provided by the activity tracker that we used in this study was the total daily steps, which is one PA parameter that reflects the user’s PA behavior, and especially bipedal locomotion. This metric is considered a reliable proxy of the overall volume of the PA performed by a subject [13]. Averaging it across a 30-day period gives us an indication of the individuals’ PA levels. The CV of daily steps is the ratio of the SD of daily steps, estimated using data recorded during a 30-day period, to the corresponding mean multiplied by 100. The CV is an indicator of the variability in PA levels and is considered here as a proxy of the regularity of the level of PA practice. A low CV value indicates low variability and therefore a high regularity of the PA level within the 30-day period used to compute the CV. Initial PA characteristics included mean PA level, which corresponds to mean daily steps during M1, and regularity or variability in PA level, which will be referred to as PA level CV. The 6-month change in PA characteristics included change in mean PA level and change in the variability of PA level.

### Assessment of Weight and Weight Change

We focused on the weight change of users over a 6-month period of use of a consumer-grade activity tracker in a real-life setting. Body weight, expressed in kg, was assessed using a connected Withings digital scale. The analyses only included individuals with at least one weight measurement within the M1 (Figure 1). The weight value closest to the beginning of M1 was used as initial body weight. Users also had to have at least one weight measurement after seven 30-day periods of activity tracker use. The weight value closest to the end of the seventh 30-day period of activity tracker use was used as the final body weight. The 6-month weight change was calculated as final weight–initial weight. This anthropometric parameter constituted the main outcome of interest.

### Statistical Analysis

In the primary analysis, multivariable linear regression models were used to explore the relationship between initial PA characteristics and 6-month weight change. PA characteristics included the mean PA level and variability in PA levels during M1. We first analyzed both the continuous mean PA level (per 1000 steps/day) and PA level CV (per 10%). We then studied a 4-category PA level classification based on Tudor-Locke classification [12,13] and the PA level CV divided into 4 quartile groups. The 4-category PA level classification consisted of sedentary (<5000 steps/day), low active (5000-7499 steps/day), somewhat active (7500-9999 steps/day), and active (≥10,000 steps/day). The sedentary category was used as the reference in the models for PA level, and for the variability in PA level, the highest quartile of CV was used as the reference category (high variability, ie, low regularity in PA level).

Multivariable models were adjusted for age category (18-30 years, 31-40 years, 41-50 years, 51-60 years, and >60 years), sex, diastolic blood pressure, systolic blood pressure, heart rate, and the predominant season during the 6-month follow-up. Blood pressure and heart rate values were assessed by computing the average of all the measures available within M1 and 6 months before M1. Study participants were categorized according to the tertile distribution of blood pressure and heart rate. Missing values for these variables were greater than 5% of the study sample; thus, missing categories were created. Indicators of PA level and PA level CV were simultaneously included in the multivariable models.

Tests for linear trends were conducted by assigning the median value of mean PA level and PA level CV to each category or quartile group and modeling this value as a continuous variable.

Multivariable restricted cubic spline regression was used to better characterize the continuous shape of the associations between mean PA level, regularity of the level, and 6-month weight change. The reference values for estimating the difference in weight change and 95% CIs were chosen as the minimum and maximum values for mean PA level and PA level CV, and 3 knots were used (the 25th, 50th, and 75th percentiles of the mean PA level and the PA level CV distributions). Therefore, restricted cubic spline regression provides us with a graphical presentation in which the y-axis represents the difference in the 6-month weight change associated with any value of mean PA level (or PA level CV) when compared with the minimum value of mean PA level (or the maximum value of PA level CV).

In a secondary analysis, both univariable and multivariable linear regression models were used to analyze the associations between the 6-month change in PA characteristics and the 6-month weight change. Changes in PA characteristics included changes in mean PA levels and in PA level variability. The change in mean PA level was studied using the continuous change (per 1000 steps/day), the change in the 4-category step-defined PA classification, and a 5-category change in mean PA level. This 5-category classification consisted of a decrease in mean PA level of more than 3000 steps per day, decrease between 3000 and 1000 steps per day, change in mean PA level between −1000 and +1000 steps per day, increase in mean PA level between 1000 and 3000 steps per day, and an increase of more than 3000 steps per day. Change in the variability of PA level included the continuous change (ie, the difference in PA level CV between M6 and M1, per 10%) and the change in quartile-based categories of PA level CV. We first defined quartile-based cut-offs at M1 and used them to categorize PA level CV at M1 and M6, and the 6-month changes between these categories were then assessed.

Statistical analyses were performed using SAS software (version 9.4; SAS Institute), and two-sided *P* values <.05 were considered statistically significant. Graphs were generated using SAS and Python (version 3.5) software.

## Results

### Characteristics of the Study Population

**Figure 2:**
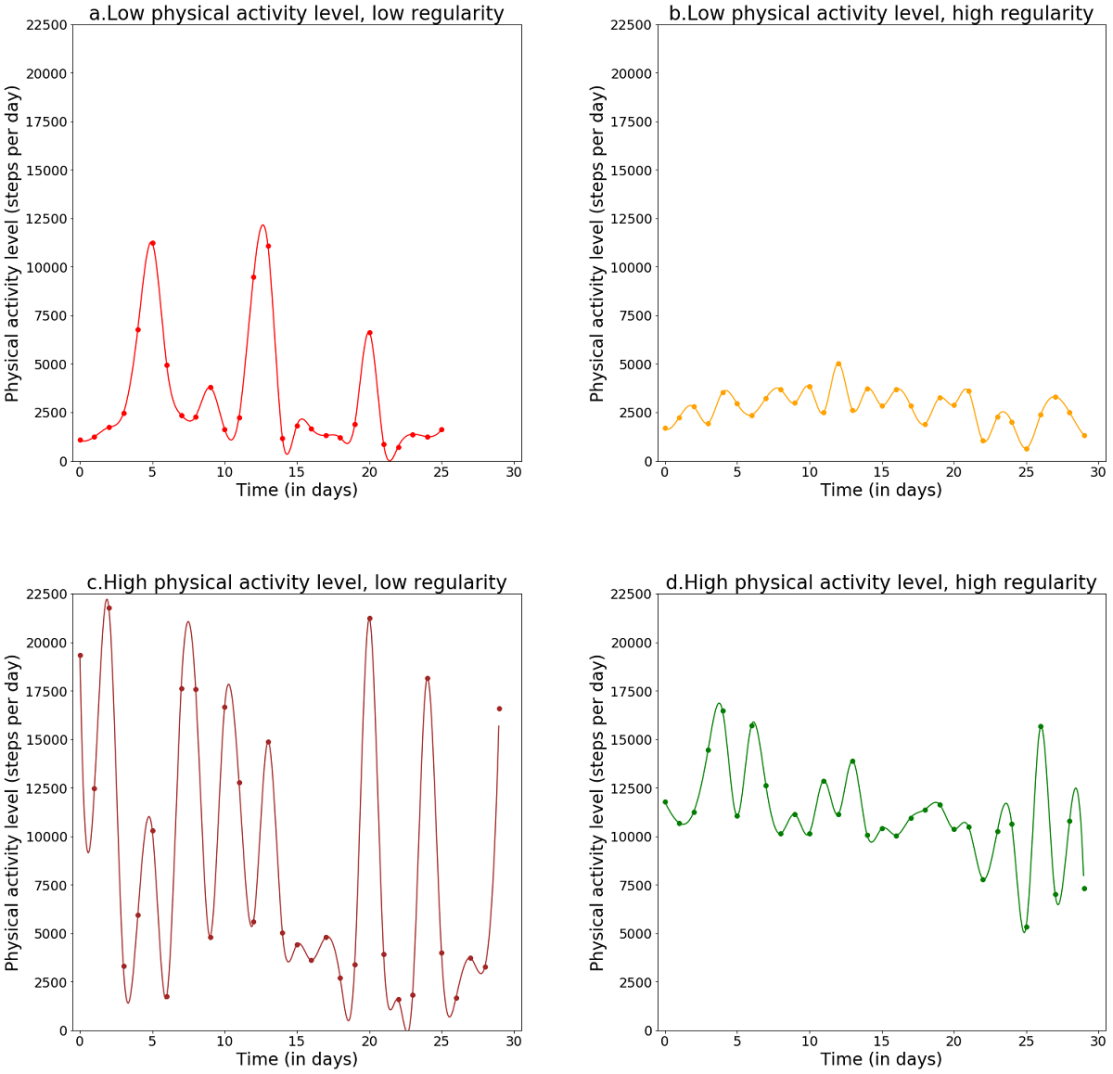
Real-word examples of evolution of physical activity (PA) level within the first 30-day period of the use of a wearable activity tracker. (a) Mean PA level=3223 steps per day; PA level coefficient of variation (CV)=98%. (b) Mean PA level=2720 steps per day; PA level CV=34%. (c) Mean PA level=8822 steps per day; PA level CV=78%. (d) Mean PA level=11,073 steps per day; PA level CV=12%.

The characteristics of the study population are displayed in Table 1 as means and standard deviations for continuous variables and as numbers and percentages for categorical variables. Individuals were described according to the initial PA characteristics based on the mean PA level and variability in PA level (PA level CV). Most users included in our sample were male (23,580/26,935, 87.54%) and aged between 41 years and 60 years (16,439/26,935, 61.03%). The initial weight in our study population had a mean value of 88.9 kg (SD 18.9). Means of PA level and PA level CV assessed during M1 were 5940.4 (SD 2929.8) steps per day and 53.4% (SD 18.9%), respectively, based on a median of 28.0 (range 2-30; Q1-Q3 23-30, IQR 7.0) step measurements per user. During the sixth 30-day period of activity tracker use (M6), the mean PA level and PA level CV were 6084.7 (SD 2977.7) steps per day and 52.1% (SD 18.6%), respectively, based on a median of 28.0 (range 2-30; Q1-Q3 21-30, IQR 9.0) step measurements per user. More than 75% of the study population had at least 20 step measurements during M1 and M6 (first quartile Q1=23 step measurements during M1 and Q1=21 step measurements during M6). Individuals in the higher step-defined PA categories tended to decrease their PA level and increase their PA level CV during the follow-up, whereas those in the lower categories tended to increase their PA level and decrease their PA level CV during follow-up. We observed similar trends in the PA level CV quartile groups, that is, individuals in the lower PA level CV quartile groups (more regular) tended to decrease their PA level and increase their PA level CV during the follow-up, whereas those in the higher quartile groups (less regular) tended to increase their PA level and decrease their PA level CV during the follow-up. Mean systolic blood pressure, diastolic blood pressure, and heart rate was 127.4 (SD 11.4) mm Hg, 79.2 (SD 8.2) mm Hg, and 70.6 (SD 10.0) bpm, respectively. Health parameters tended to be better in the higher step-defined PA categories than in the lowest category, including lower body weight, blood pressure, heart rate, and higher weight loss. However, this seemed to be the case only for systolic blood pressure and weight loss when comparing individuals in the lower quartile groups of PA level CV (more regular) with those in the highest quartile group (less regular). Figure 2 shows real-world examples of PA monitoring within the first 30-day period of the use of a wearable activity tracker for 4 users with different PA characteristics.

**Table 1:** Characteristics of the study population (N=26,935).

Variable			Overall (N=26,935)		4-category step-defined physical activity level (steps/day, M1^a^)									Physical activity level variability (CV^b^, M1)						
					Sedentary (<5000 steps/day; n=11,232)		Low active (5000-7500; n=8266)		Somewhat active (7500-10,000; n=4837)		Active (≥10,000; n=2600)		Quartile 1 (≤40%; high regularity; n=6733)			Quartile 2 (40%- 51%; n=6734)		Quartile 3 (51%- 63%; n=6735)		Quartile 4 (>63%; low regularity; n=6733)
**Age category (years), n (%)**																				
	18-30	553 (2.1)		206 (1.8)		184 (2.2)		103 (2.1)		60 (2.3)		139 (2.1)			150 (2.2)		145 (2.2)		119 (1.8)	
	31-40	3556 (13.2)		1298 (11.6)		1221 (14.8)		692 (14.3)		345 (13.3)		817 (12.1)			977 (14.5)		923 (13.7)		839 (12.5)	
	41-50	8214 (30.5)		3095 (27.6)		2678 (32.4)		1621 (33.5)		820 (31.5)		2058 (30.6)			2050 (30.5)		2112 (31.4)		1994 (29.5)	
	51-60	8225 (30.5)		3427 (30.5)		2471 (29.9)		1503 (31.1)		824 (31.7)		2007 (29.8)			2068 (30.7)		2032 (30.2)		2118 (31.5)	
	>60	6387 (23.7)		3206 (28.5)		1712 (20.7)		918 (19.0)		551 (21.2)		1712 (25.4)			1489 (22.1)		1523 (22.5)		1663 (24.7)	
**Sex, n (%)**																				
	Male	23,580 (87.5)		9589 (85.4)		7406 (89.6)		4281 (88.5)		2304 (88.6)		5854 (86.9)			5947 (88.3)		5965 (88.6)		5814 (86.4)	
	Female	3355 (12.5)		1643 (14.6)		860 (10.4)		556 (11.5)		296 (11.4)		879 (13.1)			787 (11.7)		770 (11.4)		919 (13.6)	
Baseline weight (kg), mean (SD)			88.9 (18.9)		91.7 (20.7)		88.5 (17.7)		85.9 (16.7)		84.1 (15.8)		87.5 (18.6)			89.6 (19.1)		89.7 (18.8)		88.9 (18.8)
6-month weight change (kg)^c^, mean (SD)			–1.6 (5.4)		–1.3 (5.5)		–1.6 (5.1)		–1.9 (5.3)		–2.6 (5.5)		–2.0 (5.6)			–1.7 (5.4)		–1.6 (5.4)		1.2 (5.0)
Physical activity level during M1 (steps/day), mean (SD)			5940.4 (2929.8)		3276.6 (1115.5)		6161.6 (713.2)		8593.0 (714.8)		11809.8 (1621.5)		7666.9 (3475.4)			6266.3 (2694.0)		5421.2 (2394.3)		4407.2 (1887.5)
Physical activity level CV during M1 (%), mean (SD)			53.4 (18.9)		60.7 (20.7)		53.3 (16.0)		45.7 (12.6)		36.5 (11.3)		32.7 (6.3)			45.6 (3.0)		56.4 (3.6)		78.9 (14.8)
Number of step measurements during M1^d^, median (IQR)			28.0 (23.0-30.0)		25.0 (18.0-29.0)		29.0 (25.0-30.0)		29.0 (27.0-30.0)		29.0 (26.0-30.0)		29.0 (26.0-30.0)			29.0 (25.0-30.0)		28.0 (23.0-30.0)		25.0 (19.0-29.0)
Physical activity level during M6^e^ (steps/day), mean (SD)			6084.7 (2977.7)		4203.5 (2068.8)		6232.7 (2139.8)		7984.8 (2394.6)		10206.4 (3013.2)		7364.3 (3314.5)			6341.2 (2852.6)		5705.5 (2641.9)		4928.1 (2486.5)
Physical activity level CV during M6 (%), mean (SD)			52.1 (18.6)		57.3 (20.0)		51.2 (16.6)		46.9 (15.5)		41.9 (16.0)		42.5 (15.2)			49.0 (15.3)		54.4 (17.0)		62.4 (20.5)
Number of step measurements during M6^d^, median (IQR)			28.0 (21.0-30.0)		26.0 (18.0-29.0)		28.0 (22.0-30.0)		29.0 (24.0-30.0)		28.0 (24.5-30.0)		29.0 (23.0-30.0)			28.0 (22.0-30.0)		27.0 (21.0-30.0)		26.0 (19.0-29.0)
Change in physical activity level (steps/day)^c^, mean (SD)			144.4 (2305.5)		926.9 (1948.0)		71.2 (2089.0)		−608.2 (2366.6)		−1603.4 (2759.4)		−302.7 (2372.9)			74.9 (2287.5)		284.3 (2194.7)		520.9 (2284.2)
Change in physical activity level CV (%)^c^, mean (SD)			−1.3 (20.4)		−3.4 (24.2)		−2.1 (17.9)		1.2 (15.6)		5.4 (15.2)		9.8 (15.5)			3.4 (15.4)		−2.0 (17.0)		−16.5 (22.8)
Systolic blood pressure (mm Hg)^f,g^, mean (SD)			127.4 (11.4)		128.8 (11.8)		127.2 (11.4)		125.8 (10.9)		124.5 (10.3)		126.3 (11.2)			127.2 (11.4)		127.9 (11.5)		128.1 (11.6)
**Systolic blood pressure (mm Hg), n (%)**																				
	≤122	7709 (28.6)		2831 (25.2)		2372 (28.7)		1571 (32.5)		935 (36.0)		2110 (31.3)			1990 (29.6)		1821 (27.0)		1788 (26.6)	
	122-132	7579 (28.1)		3056 (27.2)		2328 (28.2)		1404 (29.0)		791 (30.3)		1936 (28.8)			1827 (27.1)		1926 (28.6)		1890 (28.1)	
	>132	7646 (28.4)		3691 (32.9)		2322 (28.1)		1127 (23.3)		506 (19.5)		1698 (25.2)			1929 (28.6)		1985 (29.5)		2034 (30.1)	
	Unknown	4001 (14.9)		1654 (14.7)		1244 (15.0)		735 (15.2)		368 (14.2)		989 (14.7)			988 (14.7)		1003 (14.9)		1021 (15.2)	
Diastolic blood pressure (mm Hg)^f,g^, mean (SD)			79.2 (8.2)		79.9 (8.3)		79.2 (8.2)		78.4 (8.0)		77.3 (7.8)		78.6 (8.2)			79.2 (8.2)		79.5 (8.2)		79.4 (8.2)
**Diastolic blood pressure (mm Hg), n (%)**																				
	≤76	7643 (28.4)		2876 (25.6)		2336 (28.3)		1504 (31.1)		927 (35.7)		2077 (30.8)			1916 (28.5)		1820 (27.0)		1830 (27.2)	
	76-82	7640 (28.4)		3194 (28.5)		2327 (28.2)		1379 (28.5)		740 (28.5)		1891 (28.1)			1901 (28.2)		1890 (28.1)		1958 (29.0)	
	>82	7651 (28.3)		3508 (31.2)		2359 (28.5)		1219 (25.2)		565 (21.6)		1776 (26.4)			1929 (28.6)		2022 (30.0)		1924 (28.6)	
	Unknown	4001 (14.9)		1654 (14.7)		1244 (15.0)		735 (15.2)		368 (14.2)		989 (14.7)			988 (14.7)		1003 (14.9)		1021 (15.2)	
Heart rate (bpm)^f,h^, mean (SD)			70.6 (10.0)		71.6 (10.2)		70.4 (9.9)		69.8 (9.7)		68.6 (9.5)		71.0 (9.9)			71.0 (9.8)		70.7 (10.0)		69.8 (10.1)
**Heart rate (bpm), n (%)**																				
	≤66	8239 (30.6)		3071 (27.3)		2575 (31.2)		1627 (33.6)		966 (37.2)		1956 (29.1)			1962 (29.1)		2028 (30.1)		2293 (34.1)	
	66-75	8243 (30.6)		3409 (30.4)		2548 (30.7)		1484 (30.7)		802 (30.8)		2100 (31.2)			2054 (30.5)		2075 (30.8)		2014 (29.8)	
	>75	8243 (30.6)		3777 (33.6)		2477 (30.0)		1371 (28.4)		618 (23.8)		2129 (31.6)			2181 (32.4)		2083 (30.9)		1850 (27.5)	
	Unknown	2210 (8.2)		975 (8.7)		666 (8.1)		355 (7.3)		214 (8.2)		548 (8.1)			537 (8.0)		549 (8.2)		576 (8.6)	
**Predominant season during the follow-up, n (%)**																				
	Winter	6153 (22.9)		2520 (22.4)		1960 (23.7)		1098 (22.7)		575 (22.0)		1559 (23.2)			1672 (24.8)		1557 (23.1)		1365 (20.3)	
	Spring	5739 (21.3)		2326 (20.7)		1759 (21.3)		1119 (23.1)		535 (20.6)		1593 (23.7)			1517 (22.5)		1405 (20.9)		1224 (18.2)	
	Summer	7627 (28.3)		3493 (31.1)		2251 (27.2)		1216 (25.2)		667 (25.7)		1559 (23.2)			1702 (25.3)		1981 (29.4)		2385 (35.4)	
	Fall	7416 (27.5)		2893 (25.8)		2296 (27.8)		1404 (29.0)		823 (31.7)		2022 (29.9)			1843 (27.4)		1792 (26.6)		1759 (26.1)	

^a^M1: first 30-day period of activity tracker use.

^b^CV: coefficient of variation.

^c^Change=M1 value subtracted from the M6 value. Positive change indicates an increase during the follow-up.

^d^Median (IQR) are presented instead of mean (SD) due to skewed distributions.

^e^M6: sixth 30-day period of activity tracker use.

^f^Evaluated using all the data available during M1 to 6 months prior.

^g^The mean was calculated on a sample of the study population of n=22,934 because of missing data for some participants.

^h^The mean was calculated on a sample of the study population of n=24,725 because of missing data for some participants.

### PA Characteristics and 6-Month Weight Change

Associations between PA characteristics and weight changes over a 6-month period are presented in Table 2.

In multivariable analyses, we found that greater mean PA levels were inversely associated with weight change. Compared with individuals who were initially in the sedentary category (<5000 steps/day), individuals who were low active (5000-7499 steps/day), somewhat active (7500-9999 steps/day), and active (≥10,000 steps/day) had a 0.21-kg, a 0.52-kg, and a 1.17-kg greater decrease in weight (95% CI −0.36 to −0.06; 95% CI −0.70 to −0.33; and 95% CI −1.42 to −0.93), respectively. When evaluating mean PA level as a continuous variable, every 1000 steps per day increase in mean PA level was associated with a 0.11-kg decrease in weight (95% CI −0.14 to −0.09; *P* trend<.001).

Similarly, an inverse association between the regularity of PA level and 6-month weight change was observed (Table 2). The regression coefficients for weight change decreased progressively with increased regularity or reduced variability in the PA level at M1. Compared with users whose PA level CV was >63%, users whose PA level CV ranged from 51% to 63% and from 40% to 51% and whose PA level CV was ≤40% had a 0.19-kg, a 0.23-kg, and a 0.33-kg greater decrease in weight (95% CI −0.38 to −0.01; 95% CI −0.41 to −0.04; and 95% CI −0.53 to −0.13), respectively. When evaluating PA level CV as a continuous variable, every 10% increase in PA level CV was associated with a 0.07-kg increase in weight (95% CI 0.04 to 0.11; *P* trend<.001).

**Table 2:** Associations of mean physical activity level and variability in physical activity level with 6-month weight change (N=26,935).

Physical activity characteristics				Model 1^a^				Model 2^b^		
				β (95% CI) in kg		*P* value		β (95% CI) in kg		*P* value
**Mean physical activity level^c^**										
	For 1000 steps/day increase^d^			−0.13 (−0.15 to −0.11)		<.001		−0.11 (−0.14 to −0.09)		<.001
	**4-category step-defined physical activity^e^**									
		Sedentary (<5000 steps/day)	Reference		N/A^f^		Reference		N/A	
		Low active (5000-7499 steps/day)	−0.25 (−0.41 to −0.10)		.001		−0.21 (−0.36 to −0.06)		.01	
		Somewhat active (7500-9999 steps/day)	−0.60 (−0.78 to −0.42)		<.001		−0.52 (−0.70 to −0.33)		<.001	
		Active (≥10,000 steps/day)	−1.23 (−1.46 to −1.00)		<.001		−1.17 (−1.42 to −0.93)		<.001	
		*P* trend	N/A		<.001		N/A		<.001	
**Variability in physical activity level (physical activity level coefficient of variation)^c^**										
	For 10% increase^d^			0.16 (0.12 to 0.19)		<.001		0.07 (0.04 to 0.11)		<.001
	**Quartile groups^e^**									
		Q1 (≤ 40%, most regular)	−0.77 (−0.95 to −0.59)		<.001		−0.33 (−0.53 to −0.13)		.001	
		Q2 (40%-51%)	−0.50 (−0.68 to −0.32)		<.001		−0.23 (−0.41 to −0.04)		.02	
		Q3 (51%-63%)	−0.35 (−0.53 to −0.17)		<.001		−0.19 (−0.38 to −0.01)		.04	
		Q4 (>63%, less regular)	Reference		N/A		Reference		N/A	
		*P* trend	N/A		<.001		N/A		<.001	

^a^Model 1: univariable.

^b^Model 2: adjusted for age, sex, systolic blood pressure, diastolic blood pressure, heart rate, and for the predominant season during the follow-up.

^c^Evaluated during the first 30-day period of activity tracker use.

^d^Continuous variables of mean physical activity level and physical activity level coefficient of variation were simultaneously included in the multivariable models.

^e^Categorical variables of mean physical activity level and physical activity level coefficient of variation were simultaneously included in the multivariable models.

^f^N/A: not applicable.

**Figure 3:**
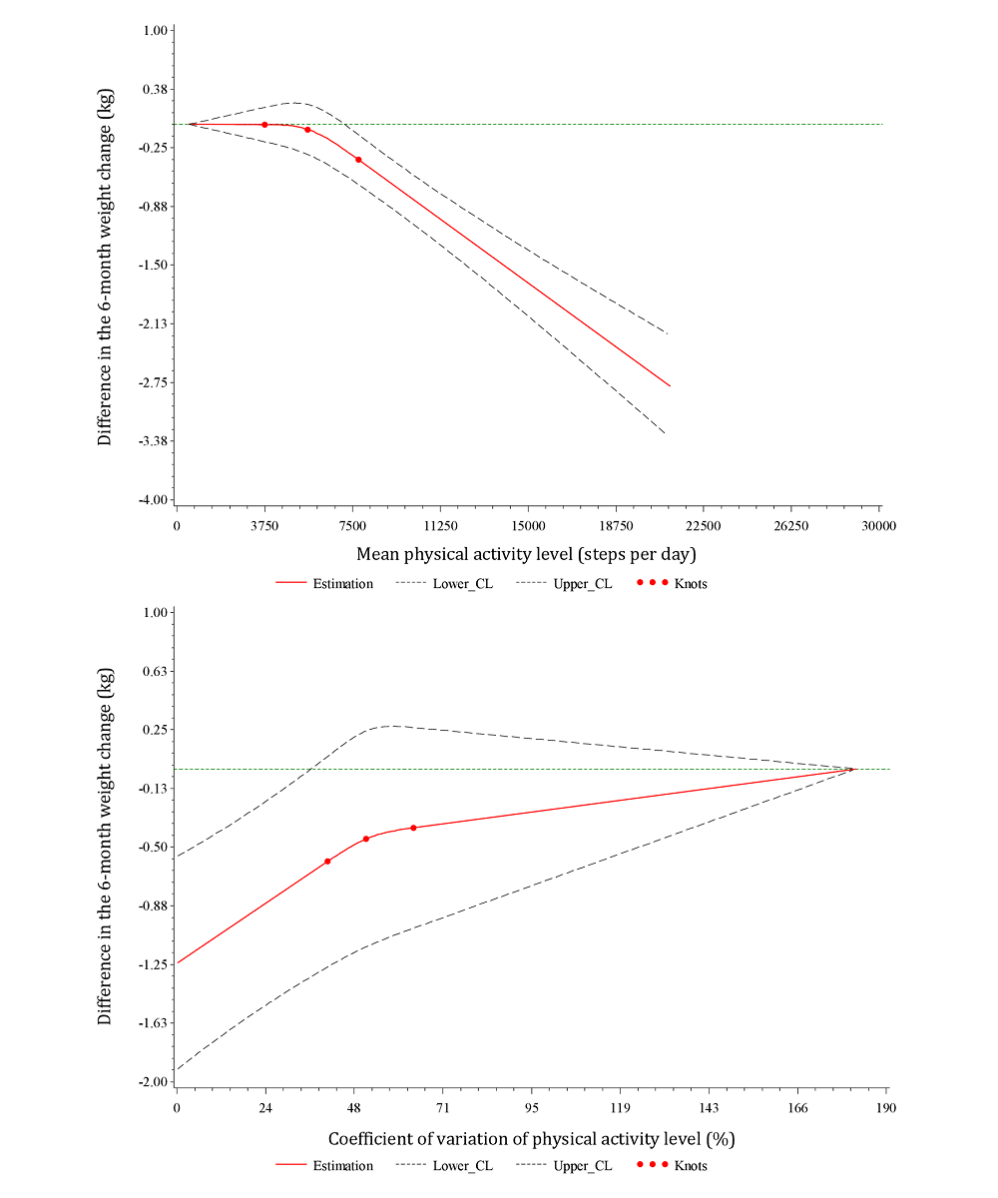
Relationships between mean physical activity level and the variability in physical activity level and 6-month weight change fitted with restricted cubic splines (3 knots placed at the 25th, 50th, and 75th percentiles). The solid red line represents the mean differences; the dashed lines are the 95% confidence limits; the green straight horizontal line corresponds to the line, y=0. The y-axis represents the difference in the 6-month weight change between individuals with any value of mean physical activity level (top) or physical activity level coefficient of variation (bottom) with individuals with the minimum value of mean physical activity level (516 steps/day; top) or with the maximum value of physical activity level coefficient of variation (182%; bottom). CL: confidence limit.

The restricted cubic spline regression analysis specified the continuous relationships between the mean PA level and regularity and the 6-month weight change (Figure 3). Figure 3 (upper part) shows a dose-response association between mean PA level and 6-month weight change, where 516 steps per day was the reference value for the mean PA level. A plateau was observed between the reference value and approximately 7500 steps per day, followed by a decrease in weight change for individuals with a mean PA level above 7500 steps per day. In Figure 3 (lower part), we observed a trend toward weight loss with an increase in regularity in PA level.

### Secondary Analysis: 6-Month Change in PA Characteristics and 6-Month Weight Change

Associations between 6-month change in PA characteristics and weight change are presented in Table 3.

An inverse association was observed between the 6-month change in mean PA level and the 6-month weight change. A 1000 steps per day increase in change in mean PA level resulted in a 0.26-kg decrease in weight (95% CI −0.29 to −0.23), whereas an increase from one category to another in the 4-category step-defined PA classification was associated with a 0.54-kg decrease in weight (95% CI −0.61 to −0.46). Compared with individuals whose change in mean PA level ranged from −1000 to +1000 steps per day, individuals whose change in mean PA level ranged from −1000 to +3000 steps per day and individuals whose change was above 3000 steps per day had a 0.68-kg and a 2.35-kg greater decrease in weight (95% CI −0.85 to −0.51 and 95% CI −2.57 to −2.12), respectively.

No association was found between the 6-month change in the variability of PA level and weight change.

**Table 3:** Associations of 6-month change in physical activity level and variability with 6-month weight change (N=26,935).

Physical activity characteristics				Model 1^a^				Model 2^b^		
				β (95% CI) in kg		*P* value		β (95% CI) in kg		*P* value
**6-month change in mean physical activity level^c^**										
	For 1000 steps/day increase		−0.28 (−0.31 to −0.25)		<.001		−0.26 (−0.29 to −0.23)		<.001	
	**5-category change in mean physical activity level**									
		Decrease of more than 3000 steps/day	−0.02 (−0.28 to 0.23)		.87		−0.10 (−0.36 to 0.15)		.43	
		Decrease between 3000 and 1000 steps/day	0.06 (−0.11 to 0.24)		.46		0.003 (−0.168 to 0.174)		.97	
		Change between −1000 and +1000 steps/day	Reference		N/A^d^		Reference		N/A	
		Increase between 1000 and 3000 steps/day	−0.73 (−0.90 to −0.56)		<.001		−0.68 (−0.85 to −0.51)		<.001	
		Increase of more than 3000 steps/day	−2.42 (−2.65 to −2.19)		<.001		−2.35 (−2.57 to −2.12)		<.001	
	Change in 4-category step-defined physical activity		−0.59 (−0.66 to −0.52)		<.001		−0.54 (−0.61 to −0.46)		<.001	
**6-month change in variability of physical activity level (change in physical activity level coefficient of variation)^c^**										
	For 10% increase		0.02 (−0.02 to 0.05)		.34		0.02 (−0.01 to 0.05)		.15	
	Change in 4-category physical activity level CV		0.03 (−0.02 to 0.09)		.21		0.04 (−0.01 to 0.10)		.09	

^a^Model 1: univariable.

^b^Model 2: adjusted for age, sex, systolic blood pressure, diastolic blood pressure, heart rate, and for the predominant season during the follow-up.

^c^Change=M1 value subtracted from the M6 value. Positive change indicates an increase during the follow-up.

^d^N/A: not applicable.

## Discussion

### Principal Findings and Comparison With the Literature

On the basis of the data from more than 25,000 connected device users, we were able to study, in free-living conditions, the associations between PA characteristics (mean level and variability), their 6-month changes, and the 6-month weight change.

Our results suggest that greater mean PA levels are associated with weight loss. Taking an additional 1000 steps per day was associated with a 0.11-kg greater weight loss over a 6-month period. In contrast with our findings, Unick et al [17] found no association between total daily steps and weight change among individuals with normal weight or overweight after 4 months, 1 year, or 2 years of a weight gain prevention intervention. However, they worked on a smaller sample of 599 participants who were predominantly female, and they compared daily steps among participants who gained >1 lb (approximately 0.45 kg) to those who lost weight or gained ≤1 lb. These differences may explain the discrepancies in the findings. Furthermore, the choice of a 1-lb cut-off point to compare participants gaining weight to those who lost weight may be too low for a 2-year intervention. In addition, Thomson et al [27] did not find any association between initial PA level (baseline mean steps/day) and 6-month weight change in a walking intervention study targeting a reduction in blood pressure. This study involved a smaller sample (179 adults) with a potentially lower variability in the studied PA profiles, which could explain the difference with our results.

When we analyzed the initial mean PA levels expressed according to the 4-category step-defined PA classification, we observed that PA levels between 5000 and 7499 steps per day, between 7500 and 9999 steps per day, and above ≥10,000 steps per day were all associated with a greater reduction in weight after 6 months in comparison with mean PA levels <5000 steps per day, which is considered as a threshold for inactivity [13,32]. Higher categories were more strongly associated with weight loss than lower categories, suggesting that “some PA is good, more PA is better” with respect to weight change. This result is in line with the study of McTiernan et al [33] that showed that higher exercise levels were linked to greater weight loss. This finding seemed logical from a purely physiological and physical point of view, that is, the greater the PA, the greater the energy expenditure, and the greater the weight loss if constant energy intakes are maintained. Our restricted cubic spline regression analysis, which was performed to better characterize the dose-response continuous relationship between mean PA level and the 6-month weight change, supported this finding. This suggests that mean PA levels above approximately 7500 steps per day were significantly associated with greater weight loss compared with the minimum value of 516 steps per day (Figure 3). Tudor-Locke et al [13] revisited the 10,000 steps per day cut-off point and proposed step-based recommendations congruent with current public health PA guidelines. These recommendations suggest that the total daily volume of PA associated with meeting the minimum recommended level is 7000 to 8000 steps per day [13,34]. Thus, our study suggests that having a PA level consistent with the current public health guidelines can be associated with weight loss. A recently published systematic review reported a dose-response relationship between daily step counts and mortality [35]. In our study, a greater decrease in weight was observed even at low levels of PA, below the commonly proposed threshold of 10,000 steps per day. Hall et al [35] showed that a reduced risk of mortality was also observed below the threshold of 10,000 steps per day.

For changes in PA levels, we observed that each 1000 steps per day increase in PA level over the 6-month follow-up was associated with a 0.26-kg greater decrease in body weight. This finding is in line with those of previous studies. Thomson et al [27] found a similar association between a 6-month change in mean steps per day and BMI change. They reported that a 1000 steps per day increase in change in PA level resulted in a 0.13-kg/m^2^ greater decrease in BMI, which corresponds to an approximately 0.38-kg greater reduction in weight considering an average height of 1.70 m [27]. This slightly stronger magnitude in the relationship could be explained by the higher initial mean PA level and mean change in PA level within the population in the Thomson et al study [27] (7279 steps/day, SD 3417 vs 5940 steps/day, SD 2930 for initial PA level and 1855 steps/day, SD 2710 vs 144 steps/day, SD 2306 for change in PA level). However, in our study, changes in PA level were evaluated by subtracting the M1 mean daily steps from the M6 mean daily steps, which is more accurate and reflects the 6-month absolute change. In contrast, in the Thomson et al study [27], the concept of change in PA was vaguer given that changes in PA corresponded to differences between steps per day values assessed during the first 2 weeks of the study and those evaluated during the remaining weeks (from the third to the 27th week). Creasy et al [36] also reported a significant association between PA level and weight change across an 18-month intervention study, suggesting an additional 0.21-kg of weight loss for each additional 1000 steps per day. Similarly, we noticed that a one-category change in the 4-category step-defined PA classification was associated with a 0.54-kg greater decrease in weight. Ganesan et al [37] found that a one-category elevation was associated with a weight decrease of 0.23 kg over a 3-month period. Similar step count categories to the 4-category step-defined PA classification were used in their study. We also observed a dose-response relationship between changes in PA level and weight change, with higher increases over time in PA level linked to greater weight loss.

In this study, we also observed that lower variability in PA levels, or greater regularity in PA levels, was significantly associated with greater weight loss. However, no association was found between the 6-month change in the variability of PA level and weight change. Even though changes in variability were not linked to weight change, the fact that the initial variability in PA level was associated with weight loss regardless of PA level suggests that step count variability can be a relevant PA component that deserves further exploration in relation to weight change and other health parameters. In addition, our results highlight the complementarity between the mean PA level and variability in PA level. Figure 2 reflects the idea that individuals with similar mean PA levels can have very different variabilities. The notion of variability in health-related indicators or behaviors has already been studied for many years in the case of glycaemia [38-41] and more recently for sleep duration [42]. These studies showed that variability in health indicators or behaviors could have harmful effects on health. This should prompt us to extend the research on variability to other health behaviors, including PA.

### Strengths and Limitations

This study has numerous strengths, including a large study population size. Indeed, it is the first of its kind to concomitantly study both PA level and variability in PA level, their changes, and weight change over a 6-month period. It is further strengthened by the fact that PA was assessed objectively via wearable activity trackers used by consumers in real-life settings and not through self-reported measures that are known to be prone to social desirability and recall bias [17]. Furthermore, most of the studies in the literature used only 3 to 7 daily step measures to assess PA of their participants during a given period [17,32,36], whereas our study population turned out to be composed of frequent users, given that 75% of the users had at least 20 daily step measures during M1 and M6. This enabled us to analyze how the variability in PA level, an indicator poorly studied in the literature, was related to weight change. Moreover, PA level was studied using the 1000 steps per day increment and the recognized 4-category step-defined PA classification proposed by Tudor-Locke et al [12,13], allowing for comparison and fostering harmonization across studies.

This study had some limitations. First, the study sample was mainly composed of men; a similar study should be conducted in another population with a larger proportion of women. The study population had a relatively high weight value at baseline (88.9, SD 18.9 kg), which may limit the generalizability of the findings to the general population. It is also possible that our findings would not be generalizable to the general population given that the individuals included in this study, consumers who chose to buy a Withings wearable activity monitor and a weighing scale, may have been more motivated to lose weight. However, we believe that any additional motivation for weight loss would be comparable with that of other populations analyzed in similar studies, such as weight loss intervention programs. In addition, the mean PA level in our study population (5940 steps/day, SD 2930) was similar to that of samples from other studies, including the Women’s Health Study cohort (5499 steps/day [34]) and the NAVIGATOR (Nateglinide and Valsartan in Impaired Glucose Tolerance Outcomes Research) study (6205 steps/day, SD 3727 [43]), showing that individuals included in our study were not particularly more active than other study populations. Information on the height of users was not available, preventing us from calculating the BMI and consequently from studying individuals who are overweight or obese specifically or from investigating if the associations observed vary by weight status. Nevertheless, we adjusted our analysis for several health-related parameters strongly correlated with BMI, such as blood pressure and heart rate. Information on food intake was not available, so we could neither control for it in our analyses nor assess the potential relative contribution of dietary intake to weight change. Finally, the intensities of the PAs performed were not accessible, as we only had access to daily step data. Nonetheless, daily step count is an increasingly widespread and intuitive metric that can be easily used as a target for health benefits. Indeed, with the expansion of wearable activity monitors and smartphones in the commercial market, the daily step count metric has become largely accessible among the general population, which justifies its use and emphasizes the need to study it in relation to health outcomes [35]. Working with data based on the number of steps accumulated during the day also implies that we only have information on ambulatory PAs [32]. Thus, participation in nonambulatory PAs, such as swimming or cycling, may not have been taken into account and properly considered. However, it is known that ambulatory PAs such as walking remain a central component of PA in the general population [35].

### Perspectives and Conclusions

Further investigations with complementary data on diet, sedentary behaviors, and PA intensity or cadence (steps/min), which will allow the calculation of time spent in moderate-to-vigorous and light PA intensities, are warranted. Alternative and/or more innovative methods such as unsupervised clustering methods (eg, k-means or hierarchical agglomerative clustering) or latent class mixed models could be used on data collected from consumer wearable devices to identify temporal evolution patterns or groups of individuals with similar behaviors and study them in relation to various health outcomes. Indeed, we also encourage our study to be replicated by focusing on other health-related parameters different from weight change such as blood pressure, heart rate, or fat mass percentage [44]. These are easily measurable with the use of connected devices and all represent key cardiometabolic risk factors to study in relation to device-assessed PA within the context of obesity epidemic and the digital revolution [44]. This study has also shown that connected devices can serve as useful tools to track PA and weight in large epidemiological studies. Future weight loss intervention studies are highly encouraged to use connected activity trackers to objectively and accurately monitor the physical behaviors of their participants. The use of activity trackers, which is increasingly becoming mainstream, should now be leveraged to track and enhance PA in order to derive truly personalized prevention programs, adapted to the individual’s lifestyle and living conditions and to move toward personalized prevention. Machine learning approaches, including neural networks, can be explored to predict, for instance, the days when a user would be less active. Such information could be used by health-related apps to send motivational messages or propose services to the users to help them become more active. More generally, we strongly promote the creation of collaborations between academic researchers and wearable device manufacturers, which could give researchers access to rich and valuable databases. Future studies on these sources of data could help improve epidemiological and health knowledge, as well as enhance health-related wearable devices.

From a public health perspective, our results suggest that weight-related health benefits can be observed below the controversial 10,000 steps per day threshold and emphasize the idea that “some PA is good, more PA is better” with regard to weight loss [11,32,35]. Our work may have important public health implications when encouraging adults to engage in PA that is monitored as steps per day, especially adults who are low active for whom adherence to the 10,000 steps per day may be too ambitious or unrealistic [34].

In conclusion, greater baseline daily step counts were associated with a greater decrease in weight. More importantly, our findings also indicate that increasing PA levels over time, irrespective of the baseline level, may be beneficial in the short term. In addition, a more regular level of PA should be promoted. Indeed, our results suggest that the variability in PA level is an interesting additional parameter to be considered as a digital biomarker candidate when assessing the impact of PA on health, which deserves to be further considered and studied in weight loss programs and observational studies. This work has shown that data from wearable devices are helpful for the digital phenotyping of large populations in real-life settings and also to suggest new metrics to characterize PA that could be easily replicated in future studies.

## Abbreviations

CV: coefficient of variation
PA: physical activity
